# Is BCG vaccination causally related to reduced COVID‐19 mortality?

**DOI:** 10.15252/emmm.202012661

**Published:** 2020-05-26

**Authors:** Masayuki Miyasaka

**Affiliations:** ^1^ Immunology Frontier Research Center Osaka University Suita Japan; ^2^ MediCity Research Laboratory University of Turku Turku Finland

**Keywords:** Immunology, Microbiology, Virology & Host Pathogen Interaction, S&S: Ethics

## Abstract

The ongoing severe acute respiratory sickness coronavirus 2 (SARS‐CoV‐2) pandemic has resulted in more than 3,600,000 detected cases of COVID‐19 illness and nearly 260,000 deaths worldwide as of May 6, 2020. Recently, BCG vaccination was shown to correlate with reduced COVID‐19 case fatality rates (preprint: Miller *et al*, 2020; preprint: Sala & Miyakawa, 2020; https://www.jsatonotes.com/2020/03/if-i-were-north-americaneuropeanaustral.html). The most recent data from publicly available resources also indicate that both COVID‐19 incidence and total deaths are strongly associated with the presence or absence of national mandatory BCG vaccination programs. As seen in Table 1, seven of eight countries with very low numbers of total deaths (< 40 per 1 million population) adopted a mandatory BCG vaccination program using one of a set of 6 separate BCG strains (Table 1). In contrast, COVID‐19 mortality was markedly higher in countries where BCG vaccination is not widely administered or is given only to high‐risk groups. COVID‐19 mortality was also higher in countries where widespread BCG vaccination was discontinued more than 20 years ago and in countries that used the BCG Denmark strain regularly or temporarily. This raises the question of whether BCG vaccination and reduced COVID‐19 mortality are causally related. An additional question is why different BCG strains may be variably associated with mortality.

## Graphical abstract

Is there more to BCG vaccination and reduced COVID‐19 case fatality rates than a mere correlation? Masayuki Miyasaka comments on this timely but intriguing question and provide some interesting points to consider.

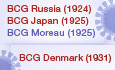

BCG (*Mycobacterium bovis* Bacillus Calmette‐Guérin) is a live attenuated vaccine for tuberculosis (TB) that is given to infants intradermally shortly after birth. In addition to protecting against TB, BCG vaccination has been shown to exert heterologous immune effects to enhance protection against unrelated pathogens (Hirve *et al*, 2012). Based on clinical findings and experimental data, BCG is hypothesized to induce sustained changes in the immune system that result in heightened responses to infections at the level of innate and adaptive immunity (Mulder *et al,* 2019; Netea *et al*, 2020). In innate immune cells, BCG induces histone modifications and epigenetic reprogramming at the promotor sites of genes encoding inflammatory cytokines such as interleukin (IL)‐1, IL‐6, and tumor necrosis factor (TNF). This process has been termed “trained immunity” (Netea *et al*, 2020) (Table 1).

**Table 1:** COVID‐19 deaths per million population and BCG vaccination

Country	Death/10^6^ a	Universal BCG vaccination program^b^	BCG strain used^b^
Spain	540	1965–1981	Denmark
Italy	478	–	–
UK	419	1953–2005	Denmark
France	381	1950–2007	Denmark
Sweden	274	1940–1975	Denmark
USA	207	–	–
Germany	82	1961–1998	Pasteur
Iran	75	+	Denmark
Finland	43	1941–2006	Denmark
Turkey	40	+	India
Norway	39	?–2009	Denmark
Korea	5	+	Multiple strains?
Australia	4	1950‐mid 1980s	Connaught
Japan	4	+	Japan
China	3	+	Russia/Bulgaria
Iraq	2	+	Japan
Taiwan	0.3	+	Japan

a Obtained from Worldometer (https://www.worldometers.info/coronavirus/).

b Obtained from Ritz and Curtis (2009) and Zwerling *et al* (2011).

The currently used BCG vaccine was initially produced at the Pasteur Institute, Paris, in 1921. The original vaccine strain was subsequently distributed to different laboratories worldwide and maintained by serial passage in each country. As shown in Fig 1, these strains can be classified as “early strains” and “late strains” depending on the timing of distribution. Notably, BCG strains that appear to be associated with lower COVID‐19 mortality (e.g., BCG Japan and BCG Russia) are both early strains, whereas BCG Denmark, which seems to induce less protection against COVID‐19, is a late strain.

**Figure 1:**
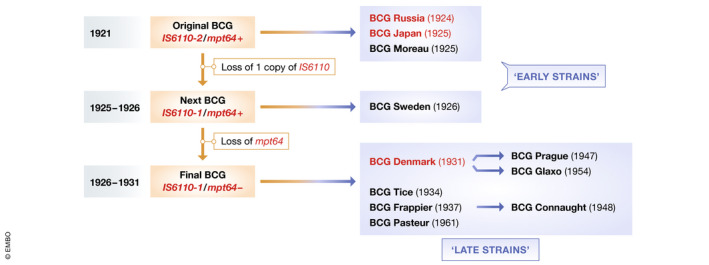
History and genetics of BCG vaccine strains The original BCG strain, first produced in 1921 at the Pasteur Institute, was distributed globally and subsequently maintained by serial passage in each country. Figure modified from Behr and Small (1999).

While one study indicated that early and late BCG strains have comparable abilities to induce delayed‐type immune responses against tuberculin (Table 2; Ladefoged *et al*, 1976), subsequent studies demonstrated that these strains differ genetically and phenotypically. First, a DNA fingerprinting study demonstrated that late strains such as BCG Denmark have evolved from early strains through genetic mutations (Behr & Small, 1999; partially depicted in Fig 1). Another study indicated that, probably because of these mutations, late strains have lost expression of several membrane proteins including MPB64, MPB70, and MPB83. Moreover, cell wall‐associated lipids including methoxy mycolate are absent in late strains, while phthiocerol dimycocerosates (PDIMs) and phenolic glycolipids (PGLs) are retained (Table 3; Chen *et al*, 2007; Liu *et al*, 2009). Another study showed that the early BCG Japan and BCG Russia strains both have much higher bacterial counts than the late strains (Table 4; WHO Technical Report, 1979), raising the interesting possibility that early strains may be richer than late strains in substance(s) that can stimulate immune responses associated with “trained immunity”.

**Table 2:** Tuberculin sensitization abilities of different BCG strains in children

BCG strains	Score
Japan	5
Pasteur	4
Denmark	4
Glaxo	2
Russia	4
Moreau	5

Data derived from Ladefoged *et al* (1976).

**Table 3:** Cell membrane composition of different BCG strains

BCG strains	MPB64	MPB70/83	Methoxy mycolate	PDIMs/PGLs
Russia	+	+++	+	+
Japan	+	+++	+	−
Moreau	+	+++	+	−
Sweden	+	+++	+	+
Denmark	−	+	−	+
Glaxo	−	+	−	−
Frappier	−	+	−	+
Pasteur	−	+	−	+

Data derived from Chen *et al* (2007) and Liu *et al* (2009).

**Table 4:** Bacterial counts in different BCG strains

BCG strains (country)	Viable bacterial counts (10^6^/ml)
Japan (Japan)	20–50
Russia (Russia)	10–30
Glaxo (UK)	8–26
Connaught (Australia)	7–15
Moreau (Brazil)	2–10
Pasteur (France)	1–10
Pasteur (Netherlands)	1–10
Denmark (Denmark)	3–7
Denmark (Germany)	1–3

Data derived from WHO Technical Report Series 638 (1979).

Two studies evaluated BCG Japan and BCG Denmark for their ability to induce cytokine secretion in peripheral blood lymphocytes. One of these, conducted in Africa, showed that BCG Japan induced more robust proliferation of CD4^+^ and CD8^+^ T cells, higher secretion of Th1 cytokines (interferon‐γ, TNF‐α, and IL‐2) and lower secretion of Th2 cytokines (IL‐4) compared with BCG Denmark (Davids *et al*, 2006). Another study conducted in Mexico showed that BCG Japan induced higher levels of IL‐1α, IL‐1β, IL‐6, and IL‐24 in peripheral blood mononuclear cells obtained from vaccinated children compared with BCG Denmark (Wu *et al*, 2007). These results suggest that BCG Japan is more efficient than BCG Denmark in inducing the production of multiple types of inflammatory cytokines.

While the exact mechanism of action remains unclear, these results collectively raise the interesting possibility that specific BCG strains such as BCG Japan may effectively induce immunity not only against *M. tuberculosis* but also unrelated pathogens. Thus, one could hypothesize that a particular BCG strain might preferentially act on the immune system in a manner that significantly reduces the morbidity/mortality associated with certain viral infections. However, data from both Finland and Australia seemingly contradict the hypothesis that early BCG strains confer resistance to COVID‐19 morbidity. These countries ceased their universal BCG vaccination programs some years ago (2006 in Finland and mid‐1980s in Australia), yet they show a low mortality of COVID‐19 per 1 million population, compared with countries with current mandatory BCG vaccination (Table 1). Thus, BCG vaccination—if it does contribute to lower COVID‐19 mortality—is clearly not the only factor. Two relevant traits shared by Finland and Australia are their excellent medical care systems and low population densities, and the latter of which could make social distancing measures more effective than in population‐dense countries.

Finally, all the abovementioned studies are observational, and no causal relationship has been substantiated yet between BCG vaccination and reduced numbers of severe and/or fatal COVID‐19 cases. While some countries have begun clinical studies to determine whether BCG vaccination can protect healthcare workers from SARS‐CoV‐2 (not yet recruiting and recruiting clinical trials, with a total of over 10,000 participants—NCT04369794, NCT04350931, NCT04362124, NCT04348370, NCT04327206, NCT04347876, NCT04328441—ClinicalTrials.gov), small‐scale clinical trials with limited numbers of participants are unlikely to provide a clear‐cut answer. This is because < 10 of 1,000 individuals are expected to become infected with SARS‐CoV‐2 even in endemic countries such as Spain, Italy, and France (with incidence of 5,285, 3,485, and 2,584 cases per million population, respectively, as of March 4, 2020). Therefore, such studies are unlikely to document a definitive effect of BCG vaccination. Human challenge studies are another possibility, but ethical issues would prohibit many of these. Instead, given that ferrets are susceptible to SARS‐CoV‐2 infection (Kim *et al*, 2020), experimental verification of this hypothesis using animal models is warranted and feasible.

## Conflict of interest

The author declares that he has no conflict of interest.
